# Correlation Between Thyroid Fine Needle Aspiration Cytology and Postoperative Histology: A 10-Year Single-Centre Experience

**DOI:** 10.7759/cureus.14504

**Published:** 2021-04-15

**Authors:** Ahmad K Abou-Foul, Jameel Muzaffar, Emmanuel Diakos, James E Best, Navid Momtahan, Sharan Jayaram

**Affiliations:** 1 Department of Otolaryngology/Head and Neck Surgery, Royal Stoke University Hospital, Stoke-On-Trent, GBR; 2 Department of Otolaryngology/Head and Neck Surgery, University Hospitals Birmingham National Health Service (NHS) Foundation Trust, Birmingham, GBR; 3 Department of Otolaryngology/Head and Neck Surgery, Walsall Manor Hospital, Walsall, GBR; 4 Department of Oncology, Worcestershire Acute Hospitals National Health Service (NHS) Trust, Worcester, GBR; 5 Department of Cellular Pathology, Royal Wolverhampton National Health Service (NHS) Trust, Wolverhampton, GBR; 6 Department of Otolaryngology/Head and Neck Surgery, Lancashire Teaching Hospitals National Health Service (NHS) Foundation Trust, Preston, GBR

**Keywords:** fine needle biopsy, thyroid cytology, thyroid cancer, diagnostic accuracy

## Abstract

Introduction

Fine needle aspiration cytology (FNAC) forms part of the routine workup for suspicious thyroid nodules. Whilst cytological analysis is less precise than histological assessment, it is quick and easy to perform and may avoid the need for invasive and potentially risky surgery.

Methods

This retrospective study spanned a 10-year period comparing preoperative FNAC with postoperative histology results to establish the accuracy of diagnosis and malignancy rates within our population. These results were then compared to the published figures in the literature.

Results

The histological reports of 659 consecutive cases of thyroid surgery between 2006 and 2015 were retrieved from our hospital database. Among the 471 patients (71.5%) who underwent preoperative FNAC, the postoperative histology was reported as benign in 352 (74.7%) and malignant in 119 cases (25.3%). Papillary thyroid cancer (PTC) was the commonest histological diagnosis. Thy1 grade was reported in 165 (30%) cases, with 19.4% having a final histological diagnosis of malignancy. In the Thy2 group, 85.3% of patients had a benign final histological diagnosis, while 14.7% had malignancy (false-negative results). Malignancy was found in 89% of Thy4 and 100% of Thy5 group patients.

Conclusions

Rates of malignancy varied considerably from those in the published literature. Each centre should be able to quote a local malignancy rate during patient counselling. It is also prudent for all units performing thyroid diagnostics to investigate the factors that might lead to inaccuracies in reporting.

## Introduction

Thyroid nodules represent a common problem for surgeons as well as a diagnostic challenge for pathologists. These can be detected in more than 60% of the general population, with an incidence of malignancy found to be around 5% [1-4]. There were 3,254 new cases diagnosed in the United Kingdom (UK) in 2017. The challenge facing clinicians dealing with thyroid nodules is to achieve an accurate preoperative diagnosis of malignancy and, therefore, fine needle aspiration cytology (FNAC) can play an important role in the diagnostic workup. It is a relatively safe, cost-effective, and simple procedure. Although it is less precise than standard histological assessment, it may help avoid invasive and potentially unnecessary surgery [1].

In current UK practice, FNAC specimens are reported according to the Royal College of Pathologists (RCPath) Thy grading system, which was first published in 2009 and revised in 2016 [1]. In its latest version, the RCPath Thy system had six diagnostic categories (DCs), as it divided the neoplasm possible category (Thy3) into Thy3a (neoplasm possible-atypia/non-diagnostic) and Thy3f (neoplasm possible, suggestive of follicular neoplasm). Thyroid cysts were also subcategorised within Thy1 and Thy2 grades as Thy1c and Thy2c, respectively. The RCPath system is well-aligned and largely comparable with the six-tiered Bethesda System for Reporting Thyroid Cytopathology (TBSRTC), and other internationally recognised reporting terminologies for thyroid FNAC [5].

Regardless of the terminology used, approximately 20% - 25% of all thyroid cytology is classified as indeterminate (Thy3a and Thy3f), in which it is not possible to differentiate between benign, malignant, or suspicious nodules [5-7]. For some of these indeterminate lesions, the current UK management guidelines recommend diagnostic removal of the affected lobe, guided by the decision of a multidisciplinary team (MDT), with potential progression to completion thyroidectomy following review of the postoperative histology [8]. This will inevitably lead to some benign lesions labeled as Thy3 or Thy4 being overtreated with hemithyroidectomy unnecessarily, with all the associated risks. Conversely, a high false-negative rate with Thy2 cytology could lead to missed cancers and potential progression of the disease. Therefore, optimising the diagnostic performance of the grading systems used in individual centres is of the utmost importance [7-8].

The aim of this retrospective study was threefold: 1) to correlate preoperative FNAC results with subsequent histology findings as the gold standard, 2) to audit the utilisation and the diagnostic performance of each category of the RCPath Thy system, including the sensitivity, specificity, accuracy, and positive predictive values for malignancy within the patient population in our locality, and 3) to compare our diagnostic performance results to previously published literature.

This article was previously posted to the Authorea preprint server on June 28, 2020, and it is not pending full publication elsewhere.

## Materials and methods

Study subjects and data acquisition

We conducted a retrospective single-centre observational study in a UK district general hospital. We included all patients who had thyroid resection (total or hemithyroidectomy) performed over a 10-year period, between January 2006 and December 2015. Eligible thyroid histology data was extracted from our digital pathology database and was correlated with FNAC where available. Data collected included age, gender, indication for surgery, details of FNAC, and the final histological diagnosis. If a patient had abnormal aspirate results taken from more than one nodule, the most abnormal result was used for analysis. If final histology reported incidental malignant lesions that were not sampled during the FNAC, these reports were excluded from the analysis.

Institutional approval of this retrospective study was waived.

Fine needle aspiration sample processing

In our institution, FNAC samples are routinely aspirated using ultrasound guidance. Each specimen contains at least an air-dried slide and an alcohol-fixed slide. Samples were prepared by the conventional methods using Papanicolaou Romanowsky-type stains. The department uses a Diff Quik Rapid Romanowsky staining kit for air-dried slides. Samples are distributed for reporting among eight general histopathologists in the department.

Data management

All FNAC data was sorted into six cytopathological groups according to the RCPath Thy grading system (Table 1) [1].

**Table 1:** Comparison Between the Royal College of Pathologists and the Bethesda Systems for Thyroid Fine-Needle Aspiration Cytology Reporting RCPath: Royal College of Pathologists; TBSRTC: The Bethesda System for Reporting Thyroid Cytopathology

RCPath Thy system	TBSRTC
Thy1: Non-diagnostic for cytological diagnosis (Thy1c: Non-diagnostic for cytological diagnosis – cystic lesion)	I. Non-diagnostic or unsatisfactory: A virtually acellular specimen or other (obscuring blood, clotting artifact, etc.)
Thy2: Non-neoplastic (Thy2c: Non-neoplastic, cystic lesion)	II. Benign: Consistent with a benign follicular nodule (includes adenomatoid nodule, colloid nodule, etc.); consistent with lymphocytic (Hashimoto) thyroiditis in the proper clinical context; consistent with granulomatous (subacute) thyroiditis
Thy3a: Neoplasm possible – atypia/non-diagnostic	III. Atypia of undetermined significance or follicular lesion of undetermined significance
Thy3f: Neoplasm possible, suggesting follicular neoplasm	IV. Follicular neoplasm or suspicious for a follicular neoplasm; specify if Hurthle cell (oncocytic) type
Thy4: Suspicious of malignancy	V. Suspicious for malignancy; suspicious for papillary carcinoma; suspicious for medullary carcinoma; suspicious for metastatic carcinoma; suspicious for lymphoma; other
Thy5: Malignant	VI. Malignant: Papillary thyroid carcinoma; poorly differentiated carcinoma; medullary thyroid carcinoma; undifferentiated (anaplastic) carcinoma'; squamous cell carcinoma; carcinoma with mixed features (specify); metastatic carcinoma, non-Hodgkin lymphoma, other

For those cytology results from the earlier years of the study, reports were ‘translated’ into Thy categories and slides were reviewed, if needed. Incidence rates of malignancy for each Thy grading and other diagnostic performance indicators were calculated and compared with figures from previously published literature and guidelines. 

Statistical analysis

The performance of thyroid cytology was assessed for its sensitivity, specificity, diagnostic accuracy, positive predictive value (PPV), negative predictive value (NPV), positive likelihood ratio (PLR), and negative likelihood ratio (NLR). For the purpose of yielding accurate results, patients with Thy1 results have been excluded when measuring the diagnostic performance of other categories and were reported separately.

## Results

In the 10-year period of our study, a total of 659 patients underwent thyroid resection. The mean age at operation was 49.6 ± 10.3 years, and 76.5% of patients were females. A total of 520 thyroid specimens were reported as benign (78.9%) and 139 (21.1%) as malignant (Table 2).

**Table 2:** Cytohistological Correlation for Malignant and Benign Cases ATC: anaplastic thyroid cancer; FNAC: fine-needle aspiration cytology; FTC: follicular thyroid cancer; MTC: medullary thyroid cancer; PTC: papillary thyroid cancer; PTMC, papillary thyroid microcarcinoma; TL: thyroid lymphoma

Thy Grading	PTC	FTC	MTC	ATC	TL	All Malignant	% Malignancy	All Benign	Total
Thy1	25 (8 PTMC)	3	0	2	2	32 (3 Thy1c)	19.4%	133 (13 Thy1c)	165 (16 Thy1c)
Thy2	23 (11 PTMC)	4	0	0	1	28 (5 Thy2c)	14.7%	163 (5 Thy2c)	191 (10 Thy2c)
Thy3a	12 (3 PTMC)	2	0	0	0	14	29.2%	34	48
Thy3f	15 (4 PTMC)	6	0	0	0	21	51.2%	20	41
Thy4	12	4	1	0	0	17	88.9%	2	18
Thy5	4	1	0	0	2	7	100%	0	7
Total with FNAC	91	20	1	2	5	119	-	352	471
No FNAC	15 (5 PTMC)	2	0	3	0	20	10.6%	168	188
Total	106 (31 PTMC)	22	1	5	5	139	24.1%	520	659

Papillary thyroid cancer (PTC) was the commonest malignancy, reported in 106 patients (76.3%), of which one-third (29.2%) were < 1 cm and were classified as papillary thyroid cancer microcarcinomas (PTMC) (Table 2). Follicular thyroid cancer (FTC) was diagnosed in 22 patients (15.8%), while medullary thyroid cancer (MTC), anaplastic thyroid cancer (ATC), and thyroid lymphoma (TL) were collectively diagnosed in under 8% of the patients (Table 2). More than 80% of the patients with benign histological diagnoses had a benign nodular goitre (N = 418), while follicular and Hürthle cell adenomas, Hashimoto’s thyroiditis, and Graves’ disease were histologically diagnosed in 68, 26, and five patients, respectively (Table 3).

**Table 3:** Benign Diagnoses at the Final Histology Reports

Type of Histology	Number of cases
Nodular Goitre	418
Follicular Adenoma (including Hurthle Cell Adenoma)	68
Hashimoto’s Thyroiditis	26
Graves’ Disease	5
Oncocytoma	3
Total	520

Correlation between the Thy grading system and histology diagnosis

Among the 471 patients (71.5%) who underwent preoperative FNAC, the postoperative histology was reported as benign in 352 (74.7%) and malignant in 119 cases (25.3%) (Table 2). Thy1 grade was reported in 165 cases, with 32 (19.4%) having a final histological diagnosis of malignancy. Around 10% (N = 16) of Thy1 lesions were cystic (Thy1c) with malignancy encountered in around 23% of these lesions. The majority of patients in the Thy2 group had a benign final histological diagnosis. However, 28 patients (14.7%) with Thy2 cytology had malignant nodules on postoperative histology (false-negative). The false-negative rate increased to 50% for the Thy2c subcategory (Table 2). Thy3a and Thy3f FNAC categories were reported in 48 and 41 patients, respectively, with histology confirming malignancy in 14 (29.2%) and 21 (51.2%) patients, respectively (Table 2). Thy4 and Thy5 categories where only recorded in 18 (3.82%) and seven (1.5%) patients, respectively, with malignancy diagnosis confirmed in 16 (89%) of Thy4 and seven (100%) of Thy5 patients. PTC was the commonest histological diagnosis in all six DCs. Interestingly, the only two patients with MTC and two out of five with TL having non-diagnostic (Thy1) FNAC and were operated upon, mainly based on clinical and radiological suspicion.

Diagnostic performance of the RCPath Thy grading system

We measured the diagnostic performance of our FNAC categories and compared the results with the most up-to-date figures published in the latest 2016 RCPath guidelines [1], and the latest meta-analyses published by Poller et al. [5-6] for the RCPath Thy system (13 articles, 3,911 nodules), and Bongiovanni et al. [9] for TBSRTC (eight articles, 6,362 nodules) (Tables 4-5).

**Table 4:** The Utilisation of Different FNAC Cytology Categories and the Implied Risk of Malignancy in Our Cohort and the Published Literature ^a^ For the RCPath Thy system ^b^ For the Bethesda System for Reporting Thyroid Cytopathology (TBSRTC). Categories I - VI correlate to Thy1 - Thy5 * Only for cases with proven histology † % ROM in Thy2 grade = PPV for malignancy ‡ PPV for benignity = NPV for malignancy FNAC: fine needle aspiration cytology; NPV: negative predictive value; PPV: positive predictive value; RCPath: Royal College of Pathologists; ROM: risk of malignancy

RCPath Thy grade	% Use of Category				% PPV for Malignancy (ROM)^*^			
	^a^RCPath guidelines [1]	^a^Poller et al. [6]	^b^Bongiovanni et al. [9]; Pooled Mean (Range)	Current study	^a^RCPath guidelines[1]	^a^Poller et al. [5];Pooled % (Range)	Current study	^b^Bongiovanni et al.[9], Pooled %
Thy1/1c	18 - 22	18 - 27	13 (1.8 - 23.6)	35	4	12 (5 - 22)	19	16.8
Thy2/2c	42 - 51	42 - 52	59 (39 - 73)	40	1.4	5 (3 - 9)^†^	15^†^ (85^‡^)	3.7^†^ (96^‡^)
Thy3a	5 - 10	5 - 10	9.6 (3 - 27.2)	10	17	25 (20 - 31)	29	15.9
Thy3f	14 - 16	7 - 14	10.1 (1.2 - 25.3)	9	Up to 40	31 (24 - 39)	51	26.1
Thy4	2 - 4	2	2.6 (1.4 - 6.3)	3.8	Up to 68	79 (70 - 87)	90	75.2
Thy5	5 - 10	2 - 7	5.4 (2 - 16.2)	1.5	Up to 100	98 (97 - 99)	100	98.6

**Table 5:** Performance of the Diagnostic Categories of the RCPath Thy System in Our Cohort ^a^ PPV for being benign in Thy2 category (Benign/[Benign + Malignant]) ^b ^NPV for being malignant in Thy3 - 5 category (Malignant/[Benign + Malignant]) CI: confidence interval; FNAC: fine needle aspiration cytology; NPV: negative predictive value; PPV: positive predictive value; RCPath: Royal College of Pathologists

	Thy3 - 5 (Any Abnormal FNAC) Value (95% CI)	Thy4 - 5 (Highly Suspicious or Malignant FNAC) Value (95% CI)	Thy5 (Malignant) Value (95% CI)	Thy4 (Suspicious) Value (95% CI)	Thy3f (Neoplasm Possible/Follicular) Value (95% CI)	Thy3a (Neoplasm Possible/Atypia) Value (95% CI)	Thy2 (Non-Neoplastic) Value (95% CI)
Sensitivity	67.8% (56.9 - 77.4)	27.6% (18.5 - 38.2)	8.1% (3.3 - 15.9)	19.5% (11.8 - 29.4)	24.1% (15.6 - 34.5)	16.1% (9.1 - 25.5)	67.8% (56.9 - 77.4)
Specificity	74.3% (68 - 80)	99.1% (96.7 - 99.9)	100% (98.3 - 100)	99.1% (96.7 - 99.9)	90.8% (86.2 - 94.3)	84.4% (78.89 - 89)	74.3% (68.1 - 80.1)
Positive Likelihood Ratio	2.6 (2.0 - 3.5)	30.1 (7.3 - 124.5)	-	21.3 (5 - 90.3)	2.6 (1.5 - 4.6)	1.0 (0.6 - 1.8)	-
Negative Likelihood Ratio	0.4 (0.3 - 0.6)	0.73 (0.6 - 0.8)	0.9 (0.9 - 1)	0.8 (0.7 - 0.9)	0.8 (0.7 - 1)	0.99 (0.9 - 1.1)	-
Positive Predictive Value	51.3% (44.6 - 57.9)	92.3% (74.3 - 98)	100%	89.5% (66.7 - 97.3)	51.2% (37.5 - 64.8)	29.2% (18.9 - 42.2)	85.3% (80.9 - 88.8)^a^
Negative Predictive Value	85.3% (80.9 - 88.8)	77.4% (75.1 - 79.6)	73.2% (71.9 - 74.4)	75.5% (73.5 - 77.4)	75% (72.6 - 77.3)	71.6% (69.3 - 73.7)	51.3% (44.6 - 58)^b^
Accuracy	72.5% (67.1 - 77.4)	78.7% (73.7 - 83.2)	73.8% (68.5 - 78.6)	76.4% (71.2 - 81.1)	71.8% (66.4 - 76.8)	64.9% (59.3 - 70.3)	72.6% (67.2 - 77.5)

Around 35% of the samples in our series were categorised as Thy1, which is higher than previously published figures (18% - 27%) [1, 6]. There were also some minor differences in the utilisation of Thy1 and Thy2 categories between the RCPath system and TBSRTC (Grade I, II), where the later system identifying fewer grade I and more grade II samples (Table 4).

While the risk of malignancy (ROM or PPV) in our Thy5 category (100%) was comparable to published literature for the RCPath system (98% - 100%) [1, 5] and TBSRTC (99%) [9], our malignancy rates were higher for all other categories (Table 4). ROM was higher for the Thy2 grade in our study (15%) compared to the RCPath system rates (1.4% - 5%) and TBSRTC (4%). ROM was also surprisingly much higher for our Thy4 patients (90%) compared to the RCPath figures (up to 68%) [1], the meta-analysis of results using the Thy system by Poller et al. (79%) [5], and the TBSRTC system meta-analysis by Bongiovanni et al. (75.2%) [9]. Combining Thy4 and Thy5 groups together (suspicious or malignant FNAC) demonstrated high specificity, PLR, and PPV for malignancy (99.1%, 30.1, and 92.3%, respectively) with low sensitivity (27.6%), moderate NPV (77.4%), and accuracy (78.7%) (Tables 4-5). Combining the Thy3 - 5 groups together (any abnormal FNAC) improved the sensitivity and the NPV (67.8% and 85.3%, respectively) at the expense of reducing the specificity, PPV, and the overall accuracy (74.3%, 51.3%, and 72.5%, respectively).

## Discussion

FNAC plays an important role in the initial evaluation and decision planning for patients with thyroid nodules. However, FNAC has drawbacks, especially with its relatively high rate of inadequate or unsatisfactory samples, necessitating repeat testing, and its inability to distinguish between benign and malignant lesions in some situations [8, 10-12]. Moreover, false-positive diagnosis of malignancy can sometimes occur, which can lead to unnecessary thyroid surgery with a 2% - 10% risk for long-term postoperative morbidity [13-14]. As the decision to pursue surgery as opposed to conservative management is greatly influenced by the FNAC results, there is a need for a consistent reporting process and rigorous evaluation of the diagnostic utility of thyroid FNAC [1, 13, 15-16].

The RCPath Thy grading system was designed to refine and improve the reporting process and to provide clarity for patient management [1]. It can provide consistent, reproducible, and auditable thyroid cytopathological reports, improve the communication process between clinicians and patients, and give figures for the predicted risk of malignancy with each cytological diagnosis [8-9].

This study builds on the growing body of literature to validate the diagnostic utility of the RCPath Thy system in guiding the day-to-day clinical management [1, 5-6]. While the validity of using six-tiered systems (like the RCPath system or TBSRTC) is justifiable by the strong reported cytohistological correlation, there was a notable variability in the implied risk of malignancy for different DCs and, subsequently, the percentage of patients undergoing surgery [1, 5-6, 9]. As standards for FNAC reporting outcomes are not universally set, quality assurance at individual institutions by undertaking regular audit is paramount to maintaining accuracy [1, 6, 17].

Our results demonstrate higher rates of malignancy and utilisation of the Thy1 non-diagnostic category in our cohort. This can be partially explained by possibly poor operator techniques [18-19]. In addition, unsatisfactory sample preparation and preservation, especially from cystic lesions, are well-recognised factors leading to higher rates of non-diagnostic aspirates [5, 8]. The Thy2 category also had a higher rate of malignancy in our cohort. Interestingly, 40% of the false-negative Thy2 cases had PTMC (< 1 cm), which can further explain the sampling challenges of smaller lesions, especially with the palpation-guided fine-needle aspiration cytology (PGFNAC) technique. In the meta-analysis performed by Wang et al., the authors noted a significant difference between the false-negative rates for benign FNAC between academic (2%) and community hospitals (10%) [20]. The authors attributed this difference to higher sampling error with palpation-guided fine-needle aspiration (PGFNA) and differences in cytological interpretation. Moreover, selection bias for treatment may also skew the ROM figures, as patients with Thy1 or Thy2 results will only undergo surgery if they show suspicious clinical or radiological features [5, 7].

Cystic changes and degenerative processes in thyroid nodules can often cause florid atypia, with a considerable potential for false-negative results and malignancy in around 14% - 17% of Thy1c and 4% - 33% of Thy2c nodules [13, 21-24]. Interestingly, when compared to the published figures, our results showed a higher ROM in Thy1c (19%) and Thy2c (50%), which can only be partially explained by treatment selection bias. However, we agree with the British Thyroid Association (BTA) guidelines that FNAC should be repeated for all Thy1 and Thy2 cases with suspicious clinical or sonographic features [8]. Table 6 summarises the recommended clinical actions for each RCPath FNAC category (modified from the British Thyroid Association Guidelines for the Management of Thyroid Cancer by Perros et al. [8]).

**Table 6:** Recommended Clinical Actions for Each RCPath FNAC Category FNAC: fine needle aspiration cytology; MDT: multidisciplinary team; RCPath: Royal College of Pathologists; US: ultrasonography Perros et al. [8]

RCPath Thy grade	Likely Recommended Clinical Action From FNAC
Thy1	Ultrasound assessment ± repeat FNAC
Thy2	Correlate with clinical and radiological (US) findings
Thy3a	Further investigation, usually US assessment ± repeat FNAC (Thy3a FNAC on repeat sample requires MDT discussion)
Thy3f	Diagnostic hemithyroidectomy
Thy4	Diagnostic hemithyroidectomy
Thy5	Therapy appropriate to tumour type, usually therapeutic surgery for papillary or medullary thyroid carcinomas (hemithyroidectomy or total thyroidectomy ± central compartment neck dissection)

One of the main aims of the RCPath Thy nomenclature is to reduce the cytological reporting variability for the indeterminate thyroid nodules. These are often challenging for clinicians and pathologists because of their heterogeneous morphology, and the difficulty to establish cytologically any invasive characteristics without thorough histopathological examination [6]. Our malignancy rates for Thy3a and Thy3f categories are notably higher, as shown in Tables 4-5. The Thy3a category can be mistakenly conceived by some cytopathologists as a ‘haven of safety', especially when assigned instead of Thy2 (to avoid false-negative results), or instead of Thy3f and Thy4 (to avoid false positive reporting and unnecessary surgery) [7].

In our cohort, Thy4 patients also had a higher malignancy rate (90%) compared to the published figures. The BTA recommendation of diagnostic hemithyroidectomy for Thy4 lesions is based on the RCPath guidelines which quote a 30% - 35% possibility of benign disease in this cohort and hence avoiding the potential long-term morbidity of total thyroidectomy [8]. However, in centres with a malignancy rate of > 90% for Thy4 cytology, an argument could be made for offering total thyroidectomy in patients with larger nodules (> 4 cm) to avoid a second procedure of completion hemithyroidectomy. Malignancy is almost always histologically confirmed in Thy5 patients, justifying our standard practice of therapeutic hemi- or total thyroidectomy ± central compartment neck dissection guided by the MDT decision [1, 8].

In our cohort, Thy4 patients also had higher malignancy rates compared to the published figures. In keeping with the BTA recommendations, our results confirm that total thyroidectomy should not be offered to Thy4 lesions as this would put at least one in 10 patients at risk of unnecessary surgery with its potential long-term morbidity (Table 6) [8]. However, malignancy is almost always histologically confirmed in Thy5 patients, justifying our standard practice of therapeutic hemi- or total thyroidectomy ± central compartment neck dissection guided by the MDT decision [1, 8].

The limitations of our study include a possibly heterogeneous population, and our study period crosses multiple revisions of the Thy system nomenclature. Since this was a retrospective study, it is sometimes difficult to ascertain that a histologically diagnosed malignant nodule is the same one aspirated for FNAC preoperatively. Moreover, we only included histologically correlated FNAC samples, which likely skewed our malignancy rates in the lower risk categories when cancer is not frequently encountered. While using a tiered classification nomenclature (like the Thy or TBSRTC systems) may improve the comparability of results between various institutions, these comparisons must be taken with caution as the results are often influenced by multiple factors. These factors include differences in thyroid cancer prevalence, variations in nodule selection for aspiration, the skill of the aspirators, the aspiration techniques, the experience of the cytopathologists, and the percentage of cases progressing to undergo surgery [6-7]. Moreover, the methods of calculating the ROMs and PPVs rates are widely variable in the literature, making it incredibly difficult to compare different studies [6-7, 16].

The other issue limiting the generalisability of the FNAC outcomes is the inherent inter- and intraobserver variability of thyroid cytology reporting [6, 13, 15, 25]. In a large multi-centre prospective study by Cibas et al. that assessed the reporting variability of the TBSRTC system, the concordance level between the local cytopathologists and a central review panel was only 64%, with 74.7% intraobserver concordance [15]. The false-positive rate of category VI (Thy5) was 6%, and these patients could potentially have undergone unnecessary surgery if they were not downgraded by the central review panel. Studies on the RCPath Thy system show a very similar pattern with the highest concordance for Thy1 and Thy5, moderate concordance for Thy2 and Thy3f, and lowest concordance for Thy3a and Thy4 categories [25].

## Conclusions

The use of tiered classification nomenclature, such as the RCPath Thy system, has paved the way to standardized thyroid FNAC reporting. However, diagnostic performance can be variable between different institutions. Our results demonstrate generally higher rates of malignancy compared to other published series. Each individual centre should be able to discuss suspicious cytology results in a multidisciplinary team setting and to be able to quote local malignancy rates during patient counseling. It is prudent for all units performing thyroid diagnostics to control the factors that might lead to reporting variability and to undertake regular audit of their performance. Adjunct immunohistochemical and molecular testing is promising and may provide a route to improve thyroid cytology outcomes in the future and thus help in standardising the reporting outcomes.
